# Non-linear leak currents affect mammalian neuron physiology

**DOI:** 10.3389/fncel.2015.00432

**Published:** 2015-11-06

**Authors:** Shiwei Huang, Sungho Hong, Erik De Schutter

**Affiliations:** Computational Neuroscience Unit, Okinawa Institute of Science and Technology Graduate UniversityOkinawa, Japan

**Keywords:** Goldman-Hodgkin-Katz equation, passive membrane properties, ionic concentration-dependence, time constant and input resistance, cerebellar Purkinje neurons

## Abstract

In their seminal works on squid giant axons, Hodgkin, and Huxley approximated the membrane leak current as Ohmic, i.e., linear, since in their preparation, sub-threshold current rectification due to the influence of ionic concentration is negligible. Most studies on mammalian neurons have made the same, largely untested, assumption. Here we show that the membrane time constant and input resistance of mammalian neurons (when other major voltage-sensitive and ligand-gated ionic currents are discounted) varies non-linearly with membrane voltage, following the prediction of a Goldman-Hodgkin-Katz-based passive membrane model. The model predicts that under such conditions, the time constant/input resistance-voltage relationship will linearize if the concentration differences across the cell membrane are reduced. These properties were observed in patch-clamp recordings of cerebellar Purkinje neurons (in the presence of pharmacological blockers of other background ionic currents) and were more prominent in the sub-threshold region of the membrane potential. Model simulations showed that the non-linear leak affects voltage-clamp recordings and reduces temporal summation of excitatory synaptic input. Together, our results demonstrate the importance of trans-membrane ionic concentration in defining the functional properties of the passive membrane in mammalian neurons as well as other excitable cells.

## Introduction

The non-voltage-gated component of excitable cell membranes, usually called the passive membrane, plays an important role in defining electrical properties of neurons. Passive membrane properties are controlled by the behavior of leak currents. Most leak channels in neurons are voltage-independent, 2-pore channels: the K^+^ permeable TASK (González et al., 2012) and Cl^−^ permeable CIC-2 channels (Jentsch et al., 2005). There is also a small contribution from TTX-insensitive, Na^+^ permeable NALCN channels (Ren, 2011).

**Figure 1:**
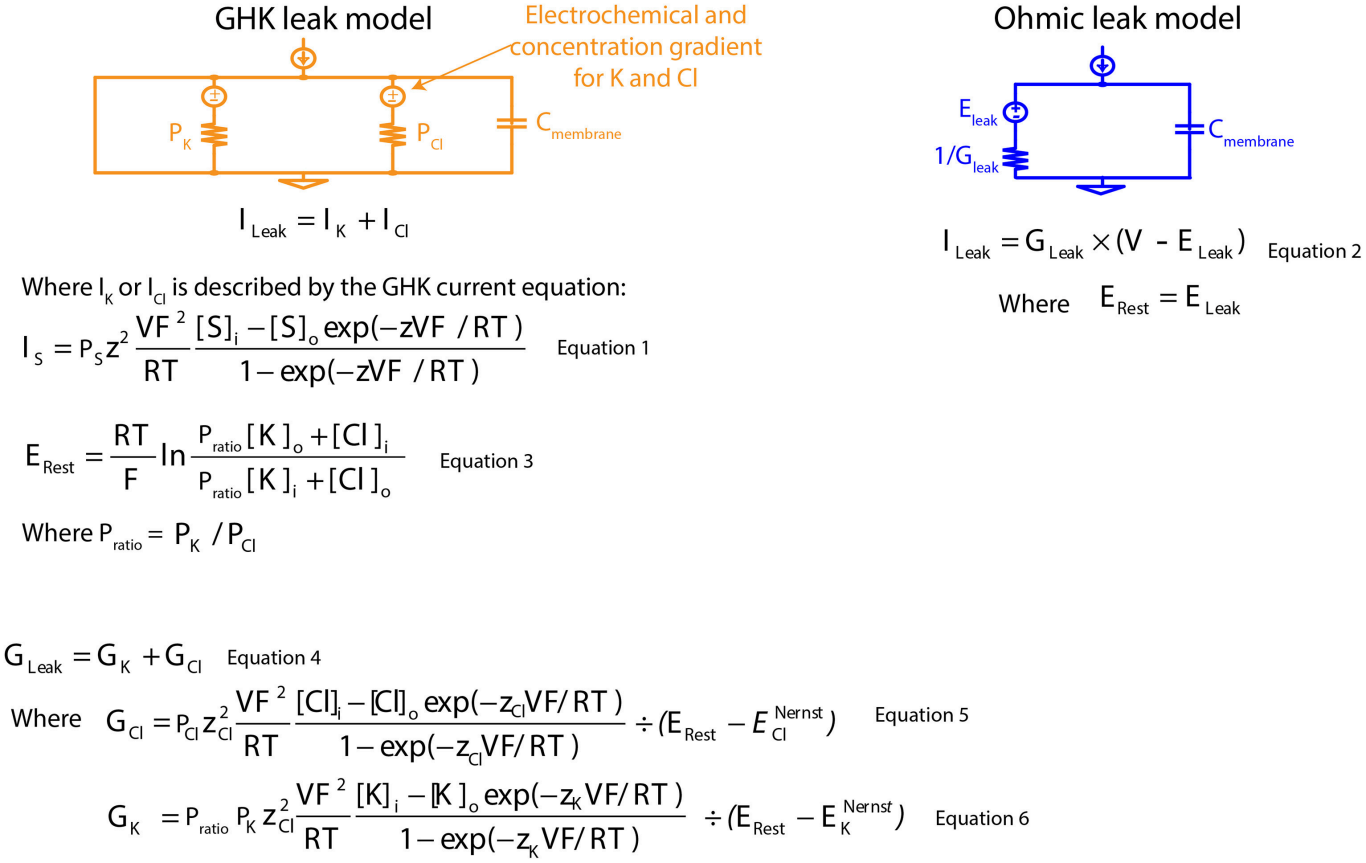
**Mathematical descriptions of the Ohmic and Goldman-Hodgkin-Katz leak-current models**. GHK, Goldman-Hodgkin-Katz; P_K_, absolute permeability value of K^+^; P_Cl_, absolute permeability value of Cl^−^; C_membrane_, specific membrane capacitance; I_Leak_, leak current; I_K_, K^+^ component of GHK leak current; I_Cl_, Cl^−^ component of GHK leak current; S, permeating ion; I_S_, GHK-type S leak current; P_S_, absolute permeability value of S; z, ionic charge of S; V, membrane potential; F, Faraday's constant; R, gas constant; T, absolute temperature; S_i_ and S_o_, intra- and extracellular concentration of S; E_Rest_, resting membrane potential; P_ratio_, permeability ratio; [K]_i_ and [K]_o_, intra- and extracellular concentration of K^+^; [Cl]_i_ and [Cl]_o_, intra- and extracellular concentration of Cl^−^; G_Leak_, membrane leak conductance; G_K_, K^+^ component of membrane leak conductance; G_Cl_, Cl^−^ component of membrane leak conductance; z_Cl_, ionic charge of Cl^−^; E^Nernst^_Cl_, Nernst potential of the Cl^−^ component of GHK leak current; z_K_, ionic charge of K^+^; E^Nernst^_K_, Nernst potential of the K^+^ component of GHK leak current; E_Leak_, reversal potential of Ohmic leak current.

Leak channels have, by definition, a voltage-independent conductance, but leak currents do show a dependence on membrane potential, as they are driven by both electrical potentials of permeating ions and ionic concentration gradients. When a concentration gradient is taken into account, the associated electrical current rectifies with the membrane potential. The degree of rectification is dependent on the concentration gradient: the greater the concentration gradient, the greater the voltage-dependent current rectification. This phenomenon is well described by the Goldman-Hodgkin-Katz (GHK) current equation (Goldman, 1943; Hodgkin and Katz, 1949; Hodgkin and Horowicz, 1959) (Figure 1: Equation 1). Nonetheless, Ohm's law, which assumes a linear relationship with membrane potential, is routinely used to describe leak currents both in experimental measurements and in neuron models (Figure 1: Equation 2).

But is Ohm's law truly a reasonable assumption of passive membrane currents? In support of a linear leak-current model, Roth and Häusser (2001) showed, in cerebellar Purkinje neurons, that two identical current pulses of opposing sign generated voltage traces that were mirror images. Furthermore, using the dual soma-dendritic patch technique, the study showed that the soma-voltage response to a current pulse in the dendrite and the dendrite-voltage response to a current pulse in the soma were superimposable when scaled. The study then used a computational model to show that the observed electrophysiological membrane properties were well modeled using a multi-compartmental neuronal model with an Ohmic leak current. However, because these data were collected at a single holding potential, any rectification due to leak currents would have been difficult to observe.

The GHK current equation has been shown to better represent voltage-gated currents than an Ohmic model. For example, voltage-gated Ca^2+^ current is much better modeled using the GHK current equation because of a 10,000-fold Ca^2+^ concentration gradient and a divalent charge (Hille, 2001). Furthermore, kinetics of voltage-gated K^+^ currents in squid axon are better modeled using GHK equations despite having a much less exaggerated concentration gradient (Clay, 1991, 1998, 2009; Clay et al., 2008).

In this study, we assess the importance of a GHK-based leak current in the passive membrane properties of single- and multi-compartmental neuronal neuron models. We show that passive membrane properties [membrane time constant (tau) and input resistance (R_n_)] modeled using Ohm's Law do not change with membrane potential; however, in a GHK-based leak model with identical membrane parameters, tau and R_n_ vary nonlinearly with both membrane potential and ionic concentration. To validate the GHK-based leak model, we investigated passive membrane properties of cerebellar Purkinje neurons in current clamp mode. We found that the relationship between tau/R_n_, membrane potential and ionic concentration, were consistent with our model predictions. Through modeling, we further show that nonlinear leak current can define the kinetics of voltage-gated ion channels and the amplitude synaptic summation. Combined, our computational and experimental evidence demonstrate the importance of non-linear leak currents in neuronal excitability.

## Materials and methods

### Design of the GHK leak model

Concentration-dependent passive membrane models are infrequently used, in part, because absolute permeability values of permeating ions (Figure 1: Equation 3) cannot be experimentally measured. We bypassed this problem by reducing the number of permeating ions modeled to two ions, and we obtained the ratio of their absolute permeability values (permeability ratio, P_ratio_) using the GHK voltage equation for a given resting membrane potential (E_rest_) (Figure 1: Equation 3). With known membrane conductance and permeability ratios, exact permeability values for both ions can be calculated (Figure 1: Equations 4–6). Modeling in this manner, allows direct comparison between linear and non-linear models, since both share the same membrane conductance and E_rest_ for current calculation.

### Modeling

All models were constructed and implemented using Python (version 2.7.5) and NEURON (version 7.4) (Carnevale and Hines, 2006; Hines, 2009) using the variable time-step method (Lytton and Hines, 2005). Unless stated otherwise, both the isopotential and compartmental models were modeled using the following membrane parameters: E_rest_ = −85 mV, specific membrane capacitance 0.8 μFcm^−2^, specific membrane resistance 120,000 Ωcm^2^, specific intracellular resistivity 120 Ωcm^2^ and a total surface area of approximately 68,065 μm^2^, with the exception of E_rest_, adapted from De Schutter and Bower (1994). Both the Ohmic and GHK membrane conductances at rest were equal to the inverse of the stated membrane resistance. Furthermore, the GHK conductance was equal to the sum of the K^+^ and Cl^−^ conductances at E_rest_.

### Channel modeling

All ion channel models were adopted from published studies. Specifically, kinetics of Kv4 and hyperpolarization-activated, non-specific cationic current were adapted from Akemann and Knöpfel (2006). Hodgkin and Huxley voltage-gated K^+^ channel current was simulated using the original model (Hodgkin and Huxley, 1952) with the original internal and external concentrations (Hodgkin, 1951). Both of these channel models were modified with GHK current instead of Ohmic I_K_, so as to investigate only the effects of non-linear leak currents. All models were created in NMODL (Hines and Carnevale, 2000) for implementation in NEURON.

### Modeling passive membrane voltage response

An Ohmic, non-specific leak current was used to describe the linear voltage-current relationship, while the combined K^+^ and Cl^−^ GHK currents were used to describe the non-linear voltage-current relationship. K^+^ and Cl^−^ absolute permeability values were calculated from the membrane conductance, the K^+^/Cl^−^ permeability ratio, and the E_rest_ using the GHK voltage equation (Equations 1, 3, and 4).

### Model validation using patch clamp electrophysiology

Mice (strain C57BL/6J 6w, Charles River) of either gender, age P17-22 and 2–4 months, were anesthetized and decapitated in accordance with the Science Council of Japan Guidelines for Proper Conduct of Animal Experiments, and with approval from the OIST Animal Resources Section.

Cerebellar sagittal slices (300 μm) were obtained at 34°C (Huang and Uusisaari, 2013; Ankri et al., 2014). Briefly, slices were obtained by dissection at 32–34°C and stored in oxygenated, artificial cerebrospinal fluid (ACSF) composed of (in mM): 125 NaCl, 2.5 KCl, 25 glucose, 25 NaHCO_3_, 1.25 NaH_2_PO_4_, 2 CaCl_2_, and 1 MgCl_2_. Slices were then transferred to a holding chamber filled with oxygenated ACSF at 34°C and allowed to recover for at least 30 min before use. Slices were discarded after 4 h. All chemicals were purchased from Sigma-Aldrich.

In the recording chamber, slices were perfused with oxygenated ACSF at 2 ml/min, 34°C. Neurons were visualized using infrared differential interference contrast video microscopy (Olympus BX51WI microscope) with a 40x water-immersion objective lens.

In whole-cell current clamp recordings, borosilicate glass electrodes of 5–7 MΩ were filled with an internal solution containing (in mM): 140 potassium gluconate, 10 KCl, 10 HEPES, 10 EGTA, 4 MgATP, 0.4 NaGTP, 10 phosphocreatine, 8 biocytin (pH adjusted to 7.3 with KOH). Purkinje neurons were identified by their distinctive morphology and position within the cerebellar cortex.

A resistance seal of ≥4 GΩ was required before entering whole-cell, patch-clamp configuration. Signals were amplified and low-pass filtered at 5 kHz using a Cornerstone BVC-700 A amplifier (Dagan), and were recorded at 40 kHz using a custom interface written in Labview acquisition software (National Instruments). After obtaining whole-cell configurations, Purkinje neurons were hyperpolarized to −65 mV.

### Isolating the passive membrane resting potential

Cerebellar Purkinje neurons were perfused with TTX (1 μM), ZD7288 (30–50 μM), and synaptic blockers (DNQX, 10 μM and GABAzine, 10 μM) to block voltage-gated Na^+^, hyperpolarization-activated cationic current (I_h_), and AMPA and GABA_a_ receptor currents, respectively. High intracellular EGTA (10 mM) was used to minimize Ca^2+^-dependent K^+^ currents, which contribute to spontaneous firing of Purkinje neurons (Edgerton and Reinhart, 2003; Khaliq et al., 2003).

We observed that the E_rest_ of cerebellar Purkinje neurons after pharmacological treatment could be permanently shifted by repeated current injections. Therefore, after each current pulse step, data were discarded if a deviation of more than 2 mV from the E_rest_ occurred. The voltage trace of each current pulse step is the average of at least four replicates.

### Analysis

Electrophysiological data were analyzed using Python 2.7, Pylab 2.7, and R 3.01. Data are given as means ± standard error and/or 95% confidence interval, and statistical significance was tested using Student's *t*-test. R_n_ was calculated from the amplitude of initial peak and steady state voltage deflection in response to a current pulse injection (−30, −15, 15 or 30 pA). Tau was estimated by a single, exponential fitting, where the fit duration was 500 ms, starting 2 ms after current pulse injection. This was done to avoid artifacts due to a voltage drop across the recording electrode (Major et al., 1994; Roth and Häusser, 2001).

## Results

Passive membrane properties of an isopotential cell are conventionally measured by applying a small current pulse to produce a membrane voltage deflection from E_rest_. From the transient proportion of the voltage deflection, tau can be estimated using an exponential decay (see Methods); from the steady-state proportion, R_n_ can be calculated. We created two leak-current models, one described by a linear circuit and the other by a K^+^ and Cl^−^ GHK current model, to compare the difference in passive membrane properties. These models are used to demonstrate non-linear properties of the GHK current model, they are not intended to fit the experimental data exactly.

### GHK leak currents make a passive membrane non-linear

**Figure 2:**
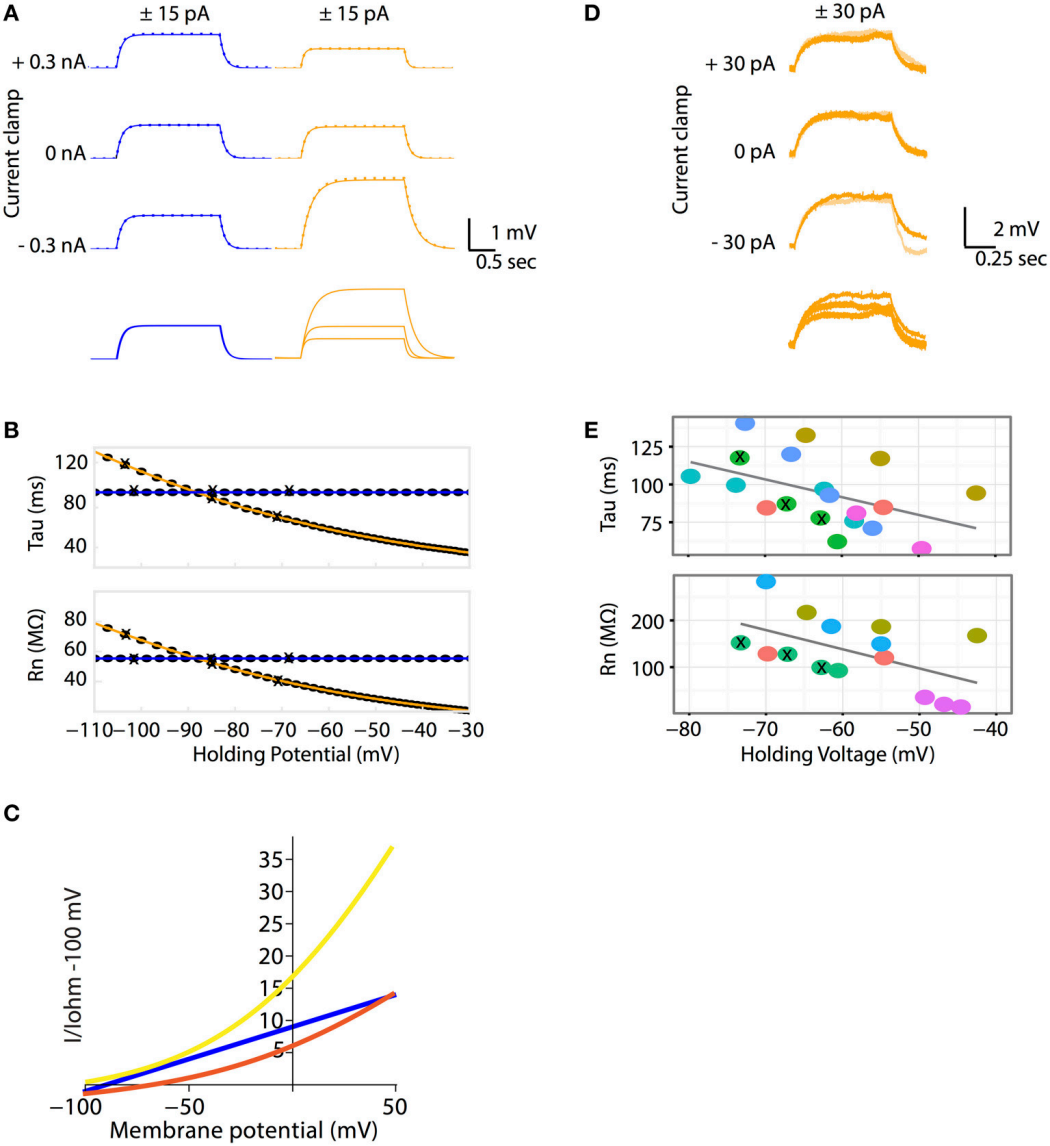
**(A–C)** The Goldman-Hodgkin-Katz leak-current model predicts non-linear voltage responses to DC current injections. **(A)** All Ohmic model simulations are colored in blue and all GHK model simulations are colored in orange. Voltage traces of passive membrane responses to +15 pA (solid lines) and −15 pA (inversed and superimposed, dotted lines) current injections for 2 seconds, at −0.3, 0 and +0.3 nA holding currents. Bottom traces: voltage responses to +15 pA current pulse at various holding currents superimposed. The Ohmic and GHK cell models have identical surface areas, E_rest_ (−85 mV) and membrane resistances (120 kohm cm^2^). For the GHK model, ionic concentrations in mM are: [K^+^]_in_ and [Cl^−^]_in_ are 150 and 10 respectively, external [K^+^]_out_ and [Cl^−^]_out_ are 2.5 and 130, respectively. **(B)** R_n_ and tau at each holding potential calculated using the +15 nA traces. The holding potential values for the traces in **(A)** are marked by “X”. **(C)** The GHK model comprises two rectifying currents I_K_ (yellow) and I_Cl_ (red). Ohmic current is colored blue. All currents are standardized to Ohmic current at −100 mV. All three current-voltage curves were constructed using the same Ohmic and GHK model as in **(A)**. **(D–E)** R_n_-voltage and tau-voltage relationships measured in cerebellar Purkinje neurons were non-linear, consistent with the GHK model prediction. **(D)** Voltage deflections in a Purkinje neuron (when major ionic currents are blocked) in response to +15 pA (orange) and −15 pA (pale orange) current pulse injections for 1 s at −30, 0, and +30 pA holding currents (corresponding holding potentials are −74, −67, and −63 mV, respectively). Bottom traces: voltage responses to +30 pA current pulse at various holding currents superimposed. **(E)** R_n_-voltage and tau-voltage relationships of each recorded cell (represented by different color filled circles). Holding potentials of the traces in **(D)** are marked by “X”. The regression slope (β) for tau and R_n_ are −1.2 (*p* = 0.043) and −4.1 (*p* = 0.037) respectively. Individual cells were highlighted to show that non-linear passive membrane properties could be observed for all cells recorded (*n* > 5).

In the Ohmic model, membrane potential changes proportionally with injected current. As a result, injecting a positive and a negative current pulse of the same magnitude (± pulses) produces two identical voltage curves, one of which is inverted. (Figure 2A). In addition, the passive membrane properties tau and R_n_, are unaffected by membrane potential change (Figure 2B). In the GHK passive membrane model, voltage deflections varied with current-clamped membrane potential. Specifically, the small current pulse that was used to produce voltage deflection in the Ohmic model, produced smaller voltage deflections at depolarizing clamped potentials and larger deflections at hyperpolarizing clamped potentials, relative to the voltage deflection at rest (Figure 2A). Notice that for any given membrane potential, the response to a positive current injection was still almost superimposable upon the response to a negative one, indicating that the response is close to linear under these conditions.

Why does voltage deflection decrease with depolarization and increase with hyperpolarization from E_rest_? In the GHK leak model, E_rest_ lies between the reversal potentials of I_K_ and I_Cl._ Therefore, I_K_ causes hyperpolarization while I_Cl_ causes depolarization. The relative proportion of the two leak currents is determined by the ratio of the degree of current rectification, determined by the concentration gradient, and their absolute permeability ratio. While in the GHK model the permeability ratio of K^+^ and Cl^−^ is close to 1, the K^+^ gradient is significantly higher than that of Cl^−^ (60-fold vs. 13-fold, respectively). As a result, K^+^ rectification is stronger than that of Cl^−^ (Figure 2C), so the effect of K^+^ hyperpolarization is stronger than that of Cl^−^ depolarization.

### Experimental verification

The crucial question addressed by this study is whether the non-linear leak model is a better fit for the passive membrane responses to electrophysiological stimulation. Using patch clamp electrophysiology in the whole-cell configuration and the current clamp protocol as described for simulations, the passive properties of cerebellar Purkinje neurons were measured. The magnitude of voltage deflections from the current injection protocol was consistent with the GHK model simulation (Figure 2D). Furthermore, tau and R_n_ decreased progressively with depolarizing potentials (Figure 2E). Individual cells were color-coded to highlight the presence of non-linear tau and R_n_ in most of the recorded cells.

**Figure 3:**
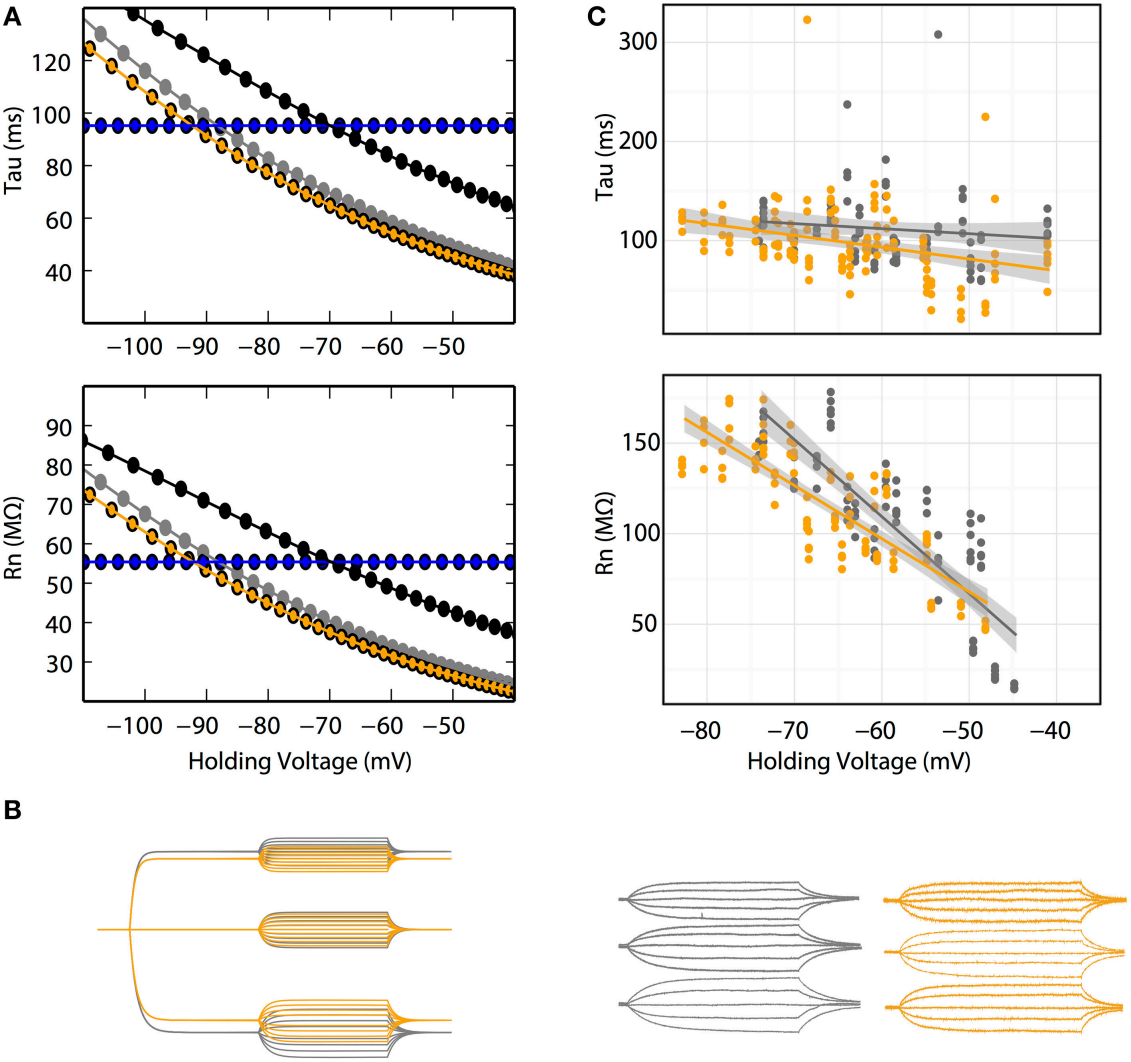
**(A)** R_n_–voltage and tau-voltage relationships are concentration-dependent in the GHK model. Voltage dependence of R_n_ and tau of 10 (gray) and 30 (orange) mM intracellular Cl^−^ at fixed E_rest_ (−85 mV). Their corresponding K^+^ and Cl^−^ permeability ratios are 1.34 and 7, respectively. Black: voltage dependence of R_n_ and tau of 30 intracellular Cl^−^ at a K^+^/Cl^−^ permeability ratio of 1.34. Ohmic simulation is shown in blue. Each data point was calculated using voltage traces from 15 pA current pulse injection. **(B)** Voltage responses to current pulses in cerebellar Purkinje neurons are consistent with the GHK model simulation. Left, GHK model simulation using a series of current pulses (−60 to +60 pA at 15 pA interval recorded at −30, 0, and +30 pA holding currents) for 10 and 30 mM intracellular Cl^−^. Right, voltage responses to current pulses (−30 to +30 pA at 15 pA interval recorded at −30, 0, and +30 pA holding currents) in cerebellar Purkinje neuron. For both simulation and experimental data, gray and orange represent 10 and 30 mM intracellular Cl^−^. Holding potentials for 10 mM Cl^−^ at −30, 0, +30 pA are identical to values reported in Figure 2E; the holding potentials for 30 mM Cl^−^ are −68, −65, and −60 mV, respectively. **(C)** R_n_–voltage and tau-voltage relationships are concentration-dependent in cerebellar Purkinje neurons. Comparisons of tau and R_n_ values at each current pulse, between 10 and 30 mM intracellular Cl^−^. 30 mM Cl^−^ reduced the steepness of the regression slope of R_n_-voltage (β_10mM_ = −5.6, *n* > 6, β_30mM_ = −2.9, *n* = 6, *p* = 0.0002).

Central to the GHK model prediction is that tau and R_n_ should be influenced by ionic concentration change. In the GHK model, changing a single ionic concentration can result in two different tau/R_n_-voltage relationships depending on which property of the model is conserved. Using intracellular Cl^−^ concentration as an example, at fixed P_ratio_, the tau/R_n_-voltage curve shifts right, toward the Cl^−^ reversal potential (Figure 3A black lines). Conversely, at fixed E_rest_, P_ratio_ increases, which increases and decreases the K^+^ and Cl^−^ leak components, respectively, and reduces the slope of the tau/R_n_–voltage relationship (Figure 3A orange lines).

In Purkinje neurons patched with 30 mM Cl^−^ -filled pipettes, smaller voltage deflections at hyperpolarizing current-clamp were observed (Figure 3B). Furthermore, the regression slope of the R_n_-voltage relationship was noticeably decreased in 30 mM Cl^−^ (β_10mM_ = −5.6, β_30mM_ = −2.9, *p* = 0.0002, *n* = 6, Figure 3C orange line) consistent with the simulation at fixed E_rest_ (Figure 3A orange line).

The effect of concentration on the tau-voltage relationship was less conclusive. The slope of the higher Cl^−^ data was steeper, contrary to our prediction (β_10mM_ = −0.5, β_30mM_ = −1.2, *p* = 0.04, *n* = 6). It is likely that this difference is not due to a physiological effect, but that it is an artifact of our estimate of tau. Voltage responses in extensive dendritic arbors, as in Purkinje neurons, have long been known to have more complex time courses than the single exponential fit used (Koch, 1999a,b; Rall, 1969). As the statistical difference is small, we suspect the effect of concentration change on tau was too small to allow experimental differentiation.

The resting membrane potentials measured under the 10 and 30 mM Cl^−^ settings were −63 ± 3 and −67 ± 2 mV, respectively, while we expected that the latter would have a more depolarized potential. The underlying mechanism of this unclear, however we suspect that it is a homeostatic response trying to maintain a set point resting potential.

### Non-linearity of passive membrane in complex morphology

**Figure 4:**
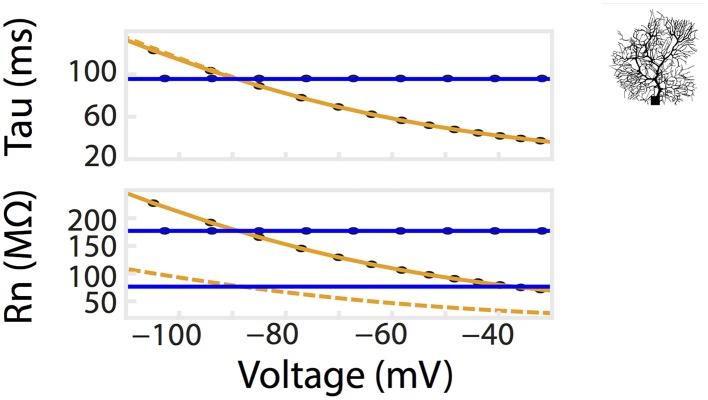
**Dendritic branching of neurons exaggerates the non-linear relationship of R_n_-voltage**. A comparison of R_n_/tau –voltage relationships was calculated from a multi-compartmental model with the same surface area as the isopotential model in Figure 2. Voltage responses of the model to a series of current pulses, as in Figure 2A. Corresponding values from simulation using the isopotential model are represented by dotted lines. Ohmic simulation results are shown in blue.

Patch clamp recordings of passive properties of mammalian neurons are routinely conducted for neuronal excitability investigations. Why hasn't passive membrane non-linearity been observed? First, as demonstrated in our simulation and whole-cell recordings, passive non-linearity is only apparent when voltage deflections from two different holding potentials are compared. At the same holding potential, the difference between positive and negative current-induced voltage traces is minimal. Second, dendritic branching increases local input resistance (Rinzel and Rall, 1974). A current injected into a branched cell would therefore result in a larger voltage deflection than that into an isopotential cell. To demonstrate this concept, we re-ran the simulation in Figure 2 using a multi-compartmental, Purkinje neuron model of the same surface area as the isopotential cell model. This showed, indeed, a much stronger dependence of R_n_ on holding potential while that of tau did not change as predicted. (Figures 2B, 4). If we had performed our patch clamp recordings in a cell type with substantially less dendritic branching, we might not have observed the concentration-dependent voltage deflections in Figure 3.

### Physiological consequences of non-linear passive membranes

**Figure 5:**
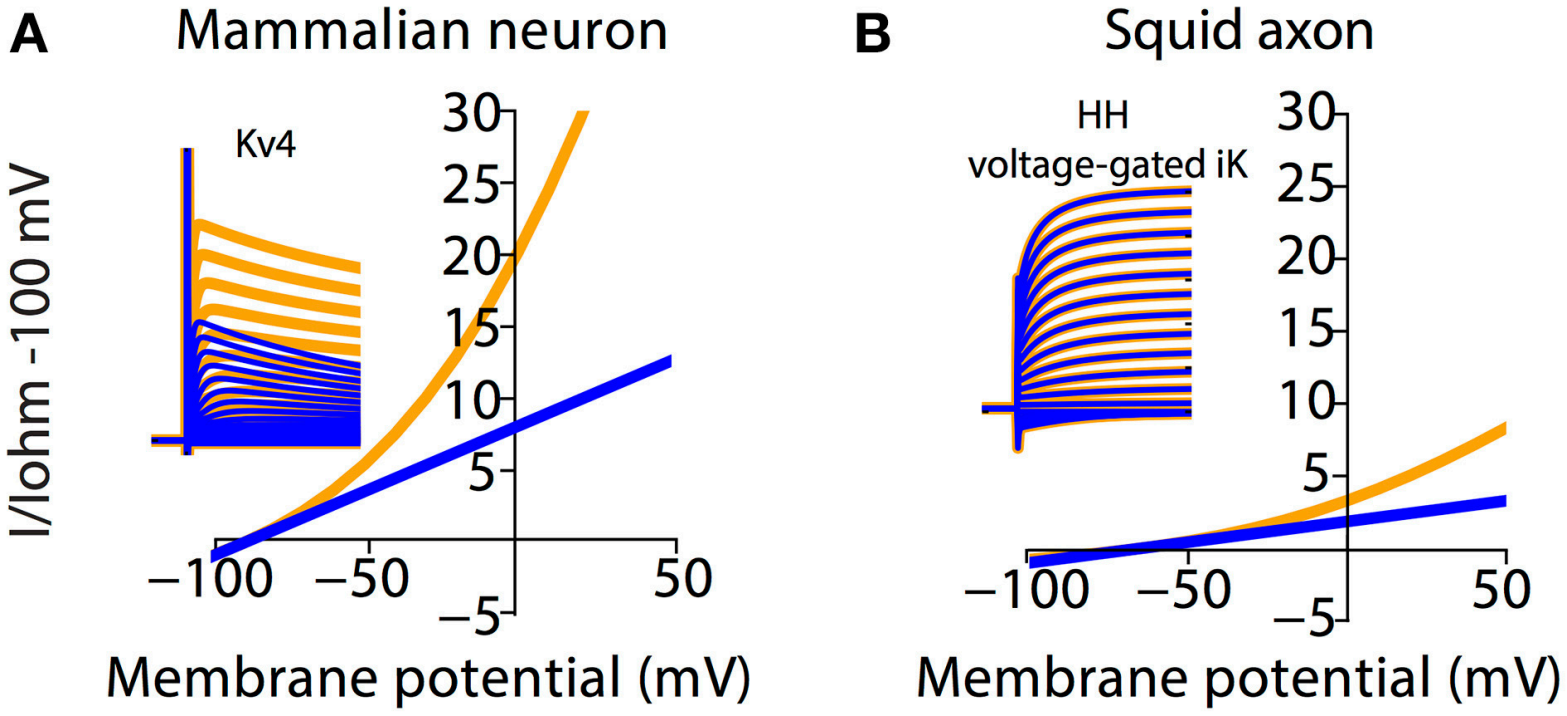
**Channel kinetic estimation using linear (Ohmic) passive membrane kinetics can cause substantial errors in cell types with large ionic concentration gradients**. Effect of GHK leak currents on simulated voltage-clamp of mammalian voltage-gated K^+^ current, Kv4 **(A)** and K^+^ current from the Hodgkin-Huxley model **(B)** The ionic concentration in **(A)** is the same as that in Figure 2. The ionic concentration in **(B)** is adapted from Table 2 in Hodgkin (1951), which listed the approximate intra- and extracellular concentration of K^+^, Na^+^, and Cl^−^ in *Loligo* axons (Hodgkin, 1951). Furthermore, E_rest_ and membrane resistance values for the squid model were set to NEURON default values. Note that both the mammalian and squid axon K^+^ currents were calculated by multiplying the gating variables with a GHK current component. Simulations using Ohmic and GHK leak currents are colored blue and orange, respectively. The initial fast transient is the capacitive current.

To illustrate the scale of errors caused by assuming an Ohmic leak instead of the GHK leak, we compared voltage-clamp simulations of the mammalian voltage-gated potassium current Kv4 under both settings (Figure 5A). It can be seen that the amplitude and rates of transient and steady state voltage properties of Kv4 differ substantially between the Ohmic and GHK model simulations. The underlying cause of this difference is the large K^+^ gradient (60-fold) in combination with the greater K^+^ permeability relative to that of Cl^−^. In contrast, in the squid axon, the K^+^ gradient is less steep (40-fold) and more importantly, the permeability ratio of K^+^/Cl^−^ is less than 1, to achieve the reported E_rest_ of −65 mV. So although K^+^ current strongly rectifies at depolarizing potentials (Clay, 2009), its contribution to the total leak is small; therefore there is little difference between the Ohmic and GHK squid axon models (Figure 5B). These examples show that, depending on the experimental preparation used, the use of Ohmic leak models may lead to significant errors in the estimate of the voltage-dependence of channel conductance from voltage clamp recordings.

**Figure 6:**
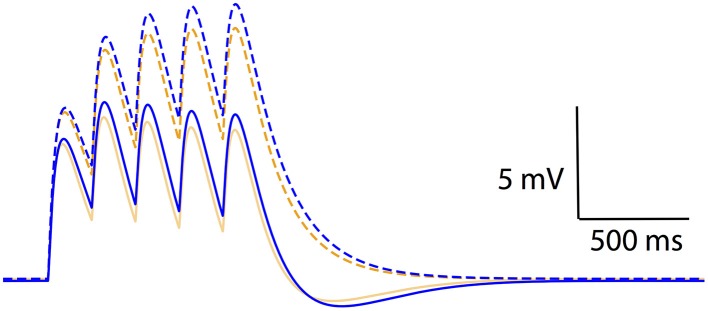
**Non-linear passive conductances affect temporal integration of post-synpatic potentials**. Temporal summation of excitatory post-synaptic potentials in an Ohmic model (blue) and a GHK model (orange) (same models as in Figure 2). Solid and dotted lines represent summation in the presence and absence of I_h_ respectively. The maximal conductance of I_h_ used in this simulation is identical to G_Leak_ of the GHK leak current.

The results thus far have dealt with non-linear voltage responses due to DC current injection; however, *in vivo* current inputs are rarely static with varying conductances and membrane potentials. We assessed the relevance of non-linear leak in this context by modeling synaptic temporal summation in a single-compartment model. Initial EPSPs were similar between the Ohmic and GHK-based leak models; however, GHK-based temporal summation was reduced relative to the Ohmic case (Figure 6, dotted lines). We also investigated temporal summation of inhibitory potentials, but the relative difference between Ohmic and GHK models was insignificant primarily because E_rest_ and the Cl^−^ reversal potential were in close proximity (not shown).

As a guide to the significance of the difference in temporal summation of EPSPs, we re-simulated this in the presence of a hyperpolarization-dependent, non-specific cationic current (I_h_) that had the same maximal conductance as that of the leak currents (see Methods). I_h_ was chosen because it is known to also dampen temporal summation (Magee, 1999; Williams and Stuart, 2000; Van Welie et al., 2006; Angelo et al., 2007). The dampening effect of GHK-leak alone on temporal summation (yellow dotted line) was small relative to that of Ih and Ohmic leak combined (blue solid line); however, when combined with I_h_ the GHK leak still had a significant effect (orange solid line). These data suggest that the effect of GHK-leak *in vivo* in the presence of voltage-gated conductances is significant.

## Discussion

Computational and experimental evidence presented here demonstrates that pharmacologically isolated passive membrane current is better modeled using the GHK than the Ohmic equation. The results suggest that non-linear leak currents are an important parameter for defining membrane electrical properties and that ionic concentration should be considered in neuronal modeling in general.

Our GHK model assumes that leak currents are generated solely from ion permeation through membrane pores, but physiological passive membranes are far from simplistic. As described earlier, leak currents are generated by specific channels, some of which may have more complex properties than those captured by the GHK model (Jentsch et al., 2005). Also, our model did not include several other membrane mechanisms that generate small currents. First, our model did not include active ion transporters like the ubiquitous Na^+^/K^+^ ATPase responsible for maintaining Na^+^ and K^+^ concentration gradients (Racker, 1978; Brodie et al., 1987) and K^+^/Cl^−^ co-transporter 2 responsible for maintaining extracellular K^+^ and intracellular Cl^−^ concentration (Payne et al., 1996; Rivera et al., 1999; Chamma et al., 2012; Seja et al., 2012). Second, the model did not take into account currents from ligand-gated ion channels, for example, tonically activated GABA_a_ currents (Brickley et al., 1996; Semyanov et al., 2004), and glutamate-gated Cl^−^ currents of excitatory amino acid transporters (Fairman et al., 1995; Tzingounis and Wadiche, 2007). Third, it did not consider volume-sensitive, outward-rectifying Cl^−^ channels, which affect both excitability and Cl^−^ flux (Inoue et al., 2005; Jentsch et al., 2005; Inoue and Okada, 2007; Zhang et al., 2011). Fourth, we did not model TTX-insensitive Na^+^ leak currents, which have been indicated in setting neuronal resting membrane potential (Ren, 2011). Some of the aforementioned molecular identities can be incorporated into a neuron model by changing the permeability ratio of a GHK-based leak model, for example, tonically active GABA_a_ currents are akin to a decrease of the K^+^/Cl^−^ permeability ratio. Other molecular identities, such as ion transporters, may need to be modeled in combination with intracellular and extracellular ion accumulation, separately from membrane leak current.

### Limitations of the modeling and experimental designs used

The primary difficulty with the GHK model is to determine the absolute permeability value for permeating ions. In our model, two out of the three most abundant physiological ions were used in the GHK model. This is so that an exact pair of K^+^ and Cl^−^ absolute permeability values could be obtained for subsequent model simulations. Ignoring the Na^+^ leak current may have caused an over-estimation of the effects of K^+^ and Cl^−^ currents, and have rendered the Cl^−^ current excitatory because the resting potential lies between the Nernst potential of the two ions. But the ability to simulate the effect of ionic concentration on E_rest_ outweighed the need to have a more complete, non-linear leak-current model. Furthermore, the consistency between our experimental and simulated data suggests that a non-linear sodium leak current would not have altered the conclusion of this study.

We used the chord conductance to calculate permeability values; however, slope conductance is equally valid for the same calculation (Thompson, 1986). The chord conductance is the slope of the line drawn from a point on a current-voltage curve to the current reversal; the slope conductance is the slope of the line tangent to the same point. Either formalism is acceptable for describing conductance as long as data acquired using either formalism are not used interchangeably (Helman and Thompson, 1982). Although permeability and conductance play similar roles in describing the dependency of channel currents on channel density, they are completely unrelated parameters and it is possible to convert one into the other only at a fixed membrane potential; see Clay (2009) for a more detailed description. As a consequence, more experimental measurements of ion permeabilities in different neuron types are needed because of their importance for passive membrane properties.

It is important to note that a cocktail of drugs was perfused to isolate the passive membrane in the experimental investigation. How these drugs affect leak channel expression during the recordings, which lasted over 40 min per cell, is unknown. Therefore, values of passive membrane properties measured experimentally may not be inherently meaningful, but relative changes in them are. This also extends to E_rest_, which slightly hyperpolarized with increasing intracellular Cl^−^ concentration, although we predicted the opposite. We suspect that this is due to cellular compensatory mechanisms to prevent depolarization from E_rest_.

### Comparisons with other studies

The main findings of this study contradict the observation of membrane linearity in cerebellar Purkinje neurons reported by Roth and Häusser (2001). It is likely that they did not observe the nonlinear leak because their study did not compare tau and R_n_ across different holding potentials. Furthermore, to observe non-linearity in their investigation of membrane reciprocity, a longer duration current pulse would have been needed.

Clay (2009) demonstrated in detail the importance of using GHK equations to model voltage-gated K^+^ channels, including the squid delayed rectifier and A-type channels. The use of GHK instead of Ohmic equations to model the delayed rectifier suffice to change the excitability of the Hodgkin and Huxley (1952) model from type 2 to type 3, with better correspondence to experimental data (Clay et al., 2008). While Clay emphasized that the need for GHK equations applies to all types of K^+^ channels, he did not consider similar effects on leak channels described here.

In somato-gastric neurons, White and Hooper (2013) reported a non-linear relationship between input resistance and input capacitance with membrane potential similar to that observed here. They speculated that it is caused by various voltage-gated ionic currents (A-type K^+^, persistent Na^+^, Ca^2+^, Ca^2+^-activated K^+^) that activate at potentials more negative than −75 mV (Turrigiano et al., 1995; Prinz et al., 2003; White and Hooper, 2013). Although our study does not rule out this possibility, it provides a simpler explanation for the non-linear properties they reported.

### Physiological consequences of membrane non-linearity

The non-linearity of passive membrane differs substantially between types of preparations, for example, it is smaller in squid axons relative to mammalian neurons. This non-linearity becomes more significant as the holding potential deviates from the resting potential of the patch-clamped cell or when the K^+^/Cl^−^ permeability ratio deviates from 1. Because electrophysiology recordings of neurons are routinely conducted under a range of voltages and the physiological K^+^/Cl^−^ permeability ratio is commonly greater than 1, the use of Ohmic leak in passive membrane modeling is almost always inaccurate.

The non-linear relationship between R_n_/tau and membrane potential, affect many routine electrophysiological measurements. If observed, they may be interpreted as evidence for the activation of voltage-gated channels by depolarization as the non-linearity has a similar potential-dependent effect on R_n_ and tau (Figure 2B; Rall, 1969; Koch, 1999a,b; White and Hooper, 2013). If present, the non-linearity will greatly affect voltage-clamp experiments (Figure 5A). The use of an Ohmic leak in the analysis of such data leads to an increasing overestimate of the conductance of the clamped ion channel as the holding potential further depolarizes, shifting the computed conductance-potential dependence to the right. In current clamp, non-linearity makes it more difficult to depolarize the membrane potential away from rest, which may explain why it is so strong in many neurons. Similarly, changes of tonic conductances at resting potential may cause smaller changes in membrane potential than predicted. Finally, the non-linearity affects physiologically relevant phenomena like temporal summation of EPSPs (Figure 6). Although *in vivo* voltage-gated channels will cause much stronger non-linearities, the effect of a GHK-based leak current is still significant.

## Conclusion

We have demonstrated that under conditions where major resting ionic conductances are blocked, passive membrane properties can change with membrane potential due to their non-linear dependence on ionic concentration. This effect is usually ignored, which leads to incorrect characterization of passive and active properties of the cell. Our results suggest that, prior to investigating active properties, it is necessary to determine passive membrane properties of the cell type of interest by fully examining the effect of ion concentration via a GHK-based current model of leak currents. Our model takes into account ionic concentration at each integration time step, which can be used to model concentration-dependent neuronal computation, as well as ion homeostasis in other excitable cells.

## Author contributions

SWH constructed models, acquired data, and analyzed both simulation and experimental data. SHH, constructed models and analyzed simulation data. SWH, SHH and ED drafted and revised the article.

## Funding

This work was supported by funding from the Okinawa Institute of Science and Technology Graduate University.

### Conflict of interest statement

The authors declare that the research was conducted in the absence of any commercial or financial relationships that could be construed as a potential conflict of interest.
