# Corrigendum: Non-linear leak currents affect mammalian neuron physiology

**DOI:** 10.3389/fncel.2015.00475

**Published:** 2015-12-10

**Authors:** Shiwei Huang, Sungho Hong, Erik De Schutter

**Affiliations:** Computational Neuroscience Unit, Okinawa Institute of Science and Technology Graduate UniversityTancha, Japan

**Keywords:** Goldman-Hodgkin-Katz equation, passive membrane properties, ionic concentration-dependence, time constant and input resistance, cerebellar Purkinje neurons

In this article, Equation (6) of Figure 1 was incorrectly written as (1), where the subscript of absolute permeability value (P) and ionic charge (z) were switched. The correct Equation is (2). This was a typo in the manuscript; all simulations presented in the original paper were performed with the correct equation.

(1) $${\text{G}}_{\text{K}}={\text{P}}_{\text{ratio}}{\text{P}}_{\text{K}}{\text{z}}_{\text{Cl}}^{\text{2}}\frac{{\text{VF}}^{\text{2}}}{\text{RT}}\frac{{\left[\text{K}\right]}_{\text{i}}-{\left[\text{K}\right]}_{\text{o}}\text{exp}(-{\text{z}}_{\text{k}}\text{VF/RT})}{\text{1-exp}(-{\text{z}}_{\text{k}}\text{VF/RT})}÷\;({\text{E}}_{\text{Rest}}-{\text{E}}_{\text{K}}^{\text{Nernst}})$$

The correct equation is

(2) $${\text{G}}_{\text{K}}={\text{P}}_{\text{ratio}}{\text{P}}_{\text{Cl}}{\text{z}}_{\text{K}}^{2}\frac{{\text{VF}}^{2}}{\text{RT}}\frac{{\left[\text{K}\right]}_{\text{i}}-{\left[\text{K}\right]}_{\text{o}}\text{exp}(-{\text{z}}_{\text{k}}\text{VF/RT})}{\text{1}-\text{exp}(-{\text{z}}_{\text{k}}\text{VF/RT})}÷\;({\text{E}}_{\text{Rest}}-{\text{E}}_{\text{K}}^{\text{Nernst}})$$

## Conflict of interest statement

The authors declare that the research was conducted in the absence of any commercial or financial relationships that could be construed as a potential conflict of interest.

